# Pain Lateralization in Trigeminal Neuralgia

**DOI:** 10.5812/aapm.3448

**Published:** 2012-07-10

**Authors:** Mark Obermann

**Affiliations:** 1 Department of Neurology, University of Duisburg-Essen, Essen, Germany

**Keywords:** Pain, Trigeminal Neuralgia, Foramen Ovale

Dear Editor,

I read the article by Bangash (1) with great interest, as it reconfirms the repeatedly reported finding of the predominance of affection of the right side of the face in patients with trigeminal neuralgia. This finding is even more intriguing as trigeminal neuralgia behaves quite opposite to many other painful disorders as pain in general was assumed to be more common on the left side of the body (2). This phenomenon was first reported in 1859 (3) and is based on the assumption that the left and right hemispheres differ in their capacity to integrate and discriminate sensory input as well as a proposed dominance of the right hemisphere for emotion processing (2). However, the lateralization of pain was not found in successive studies that tried to reconfirm these findings, so that the assumption of a lateralized pain perception in general could not be sustained (4).

It is interesting that most systematic clinical evaluations on trigeminal neuralgia report a predominance of the right side of the face to be affected by the disease. This is contrary to the left dominance theory and was explained by a lack of neural crossover in the facial region in regard to chronic facial pain in general (5, 6). These assumptions were discarded later as newer studies were unable to reconfirm this finding in all facial pain disorders (6). However, in trigeminal neuralgia the finding of lateralization persists and is reconfirmed by the study of Bangash. A recent population based study showed similar result with 60% of confirmed trigeminal neuralgia cases were affected on the right side (7). In this study female predominance (70%) was also reported, which is in line with the study by Bangash (7). Unfortunately, the genuine pathophysiologcial origin of this phenomenon remains unknown. It was hypothesized that a narrower foramen rotundrum and foramen ovale on the right side might be responsible for this lateralization, but valid scientific evidence that would support these assumptions is still lacking. Until a sound explanation is found, we have to settle for the mere description of this phenomenon and will see whether it stands the test of time or whether it will be cleared as larger patient populations will be examined or reevaluated.
